# CFD simulation of air layer effects on RT42 PCM melting in a square cell

**DOI:** 10.1038/s41598-025-16573-6

**Published:** 2025-08-25

**Authors:** Karrar A. Hammoodi, Walaa Nasser Abbas, Ali Habeeb Askar, Mohammed Azeez Alomari, Ahmed M. Hassan, Hasan Qahtan Hussein, Abbas Fadhil Khalaf, Mujtaba A. Flayyih, Saif Ali Kadhim

**Affiliations:** 1 College of Engineering, University of Al Maarif, Al Anbar, 31001 Iraq; 2 Petroleum Engineering Department, College of Engineering, University of Kerbala, Karbala, 56001 Iraq; 3 Air Conditioning Engineering Department, Faculty of Engineering, Warith Al- Anbiyaa University, Karbala, 56001 Iraq; 4 Department of Fluid and Heat Engineering, University of Miskolc, Miskolc, Hungary; 5 Institute of Physics and Electrical Engineering, University of Miskolc, Miskolc, Hungary; 6 College of Mechanical Engineering, University of Technology- Iraq, Baghdad, Iraq; 7 Department of Mechanical Engineering, University of Al-Qadisiyah, Al-Diwaniyah, 58001 Iraq; 8 Faculty of Engineering, Kerbala University, Karbala, 56001 Iraq; 9 Prosthetics and Orthotics Engineering Department, College of Engineering and Technologies, Al-Mustaqbal University, Hillah, 51001 Iraq

**Keywords:** Air layer, CFD, Melting process, PCMs, Square cell, ANSYS/FLUENT, Energy science and technology, Energy storage, Mechanical engineering

## Abstract

Latent heat storage technology has been receiving significant attention from scientists, researchers, and engineers working in solar heating and cooling, waste heat recovery, as well as building energy management. Phase change materials (PCMs) have been extensively utilized for this purpose due to their high energy storage capacity and cost-effectiveness. In this study, a numerical investigation was conducted to evaluate heat transfer in paraffin wax RT42 during its complete phase transition from solid to liquid within a square cell, both with and without an air layer on the left hot wall, with the rest of the walls thermally insulated. The enthalpy-porosity approach was quantitatively analysed using the ANSYS/FLUENT 16 program. The results indicate that the presence of a 1 mm thick air layer doubled the complete melting time, and a 2 mm thick air layer tripled the melting time compared to scenarios without an air layer. Additionally, it was shown that thermal conduction drives early melting, while density differences influence free convection in later stages. This study underscores the significant impact of air layers in delaying the melting process of PCMs paraffin wax in square latent heat storage units. Furthermore, guidelines for future investigation were provided, including examining the effects of adding air layers and providing a heat flow from the top or bottom of the square cell. This further research might assist in revealing more specifics of the interactions among the environment, phase change mechanisms, and heat transport in thermal energy storage systems.

## Introduction

The rising importance of energy and its thorough penetration into daily human activity have significantly shaped modern civilisation. On the one hand, some energy sources are necessary for supporting the development of industrialisation and technology, but they are also major potential environmental hazards that are finite. Facing such obstacles, researchers worldwide actively explored renewable energy solutions to ensure a consistent and reliable energy supply. Renewable energy is cost-effective in the long term, with reduced environmental impact and carbon footprint. By capturing power from emerging sources like solar, wind, and geothermal power, communities can transition towards a more sustainable and pleasant energy future while decreasing the harmful outcomes associated with traditional fossil energy sources. The intermittent nature of renewable resources, such as wind and solar radiation, poses challenges for consistent energy conversion^1–4^. This limitation prompted researchers to look into advanced energy storage technologies to provide a steady energy supply^5–7^. Thermal energy storage happens to be one of the best options to deal with this issue because phase change materials (PCMs) have very high storage capacities and are highly cost-effective^8,9^. These materials have attracted great attention for their efficient storage and release of thermal energy PCMs have been applied in different areas including solar heating-cooling systems^10–12^ and building energy management strategies^13^. For enhanced efficiency during thermal storage, PCMs are generally encased in containers or molds in different geometrical configurations such as cylindrical, rectangular and spherical shapes^14–16^. The type of encapsulation design significantly influences heat transfer performance and thermal stability of the storage system.

Multiple research studies have investigated phase change material (PCM) thermal performance in square containers through experimental and numerical methods. A complete analysis of pre-saturated n-octadecane (PCM) with CuO nanoparticle dispersion in a square container was performed by Dhaidan et al.^17^. Experimental measurements together with computational analysis helped them assess heat transfer characteristics. The experimental measurements took temperature readings from various positions inside the container as insulation covered the front, top and left walls yet the right wall received heat exposure. A finite element method served as the numerical solution technique to resolve the equations of continuity, momentum and energy. The research investigated three essential parameters which consisted of sub-cooling effects together with Rayleigh number and nanoparticle concentration. Experimental findings matched theoretical calculations to a high degree. A higher concentration of nanoparticles in PCM enabled faster heat transfer and shorter charging times because it increased PCM temperature and accelerated heat distribution within the container. The use of high nanoparticle concentrations demands extra attention because it produces thick liquid and may trigger particle clustering. Heat transfer in the lower section of the container operated through conduction yet free convection controlled the upper area. Charging time decreased as the Rayleigh number increased. Additional sub-cooling increases the melting process duration by extending the charging period according to the study findings. Kean et al.^18^ reviewed ANSYS Workbench 17 in a numerical study. The research studies the paraffin wax melting behavior when nanoparticles are introduced to a square container. The authors also investigated the thermal effect of the side wall and the distribution in the phase change material (PCM) of the nanoparticles. By their analysis, they estimated higher heat transfer rates with low-concentration nanoparticles and showed that heating from the side wall of the container was superior to base heating in terms of melting rate. However, some authors have investigated the thermal performance of Phase Change Materials (PCMs) in square cavities through experimental and numerical methods, considering different influence factors. Korti and Guellil^19^ studied the influence of the inclination angle of the square container on the melting of PCM. Their study was for vertical (0°), inclined (45°), and bottom horizontal (90°) positions, showing inclination has a significant effect on free convection, heat transfer rate, and melting front. The results demonstrated that the bottom-horizontal and inclined cases comprised 56% and 48% decreased melting times, respectively, when compared to the vertical configuration, with enhanced free convection involved in the bottom configuration. Following the influence of heating conditions, Bhattacharjee et al.^20^ studied theoretically PCM melting behavior under several wall heating cases. They noticed that bottom wall heating from the beginning resulted in a faster melting than side wall heating. However, as melting proceeded, side-wall heating increased convective heat transfer because of the larger solid-liquid interface, while top-wall heating led to thermal stratification, suppressing convection. Ho et al.^21^ investigated by computational analysis and demonstrated that suspensions of PCMs in water enhanced the heat transfer by free convection by as much as 70% compared to water alone. An additional key factor influencing PCM thermal performance is the enclosure’s tilt angle that was studied by Rashid et al.^22^ using ANSYS-FLUENT 16 software. The study showed that tilting a horizontal square enclosure of 15°, 30°, or 45° raised the melting time by 15%, 42%, and 71%, respectively. Besides, the highest PCM temperature was found in a horizontal position (352 K) but reduced to 332.5 K when initially tilted at 45°. The combination of these studies gives an overall insight into the mechanisms of PCM melting and solidification in square cavities. They highlight the importance of inclination angle, heating location, aspect ratio, fin shape, and tilt angle in achieving high thermal performance^23–26^.

Many researchers have recently been investigating various parameters and cell shapes to improve the energy storage efficiency in PCM-based systems. Hammoodi et al.^27^ utilized CFD simulations in order to investigate the influence of air layers on the melting processes for phase change material (PCM) in the spherical and semi-cylindrical containers. The authors sought to research the effects of air voids on the heat transfer, and phase change behavior of latent heat storage systems. The study demonstrates that air layers increase the thermal insulation and therefore prolong heat load and consequently melt delay, the delay time being more pronounced at lower density values. Cooling was in excess of the measured reference as a result of heat transfer mechanism interference by air pockets from phase change material to the surrounding environment causing cooling lateral gradients. The study showed how geometric configurations influence the air layers during the melting process. Spherical vessels exhibited a continuous thermal resistance effect due to the air layer, while semi-cylindrical vessels displayed isolated thermal resistance regions, resulting in prolonged melting time. The findings indicate that designs of PCM-based thermal storage systems ought to incorporate an air layer to enhance heat transmission. In their study, Kadhim et al.^28^ look into how to improve the charging capacity of RT42 paraffin wax in a square enclosure (50 × 50 mm) that has copper fins of different lengths and also orientations. Each cell utilized four fins, each with a uniform thickness of 1 mm. The fin lengths of each cell were 10, 20, and 30 mm, which accounted for the discrepancy. A heat flux-maintained steadiness on the wall containing fins, while other walls received insulation treatment. Among the tested cells, the longest 30 mm fins achieved the highest charging rate because they yielded a performance improvement of 55.56% compared to unfurnished cells. Placing the fins inside one of the insulated walls caused a reduction in charging speed since the fins should be positioned directly toward the thermal charging source.

Following an extensive review of the literature on the thermal performance of PCMs during the melting process, it is evident that prior studies have extensively examined various influential parameters—such as the direction of heat application, the use of fins, or the inclusion of nanoparticles. However, no research has specifically investigated the effect of a static air layer on the overall melting duration of PCM in square enclosures. Prior works such as Hammoodi et al.^27^ and Khalaf et al.^29^ examined the role of air layers in non-square geometries, such as hemispherical and semi-cylindrical enclosures, while Kadhim et al.^28^ focused on enhancing heat transfer in square cells using internal fins. In contrast, the current study offers a novel numerical analysis of the melting behavior of RT42 paraffin wax inside a square cavity with 1 mm and 2 mm thick static air layers on the heated wall. Using ANSYS/FLUENT 16 and the enthalpy-porosity method, this work quantifies the impact of air gaps on thermal resistance and melting delay—providing original insights relevant to square latent heat storage systems.

## Numerical analysis

### Mathematical models

**Fig. 1:**
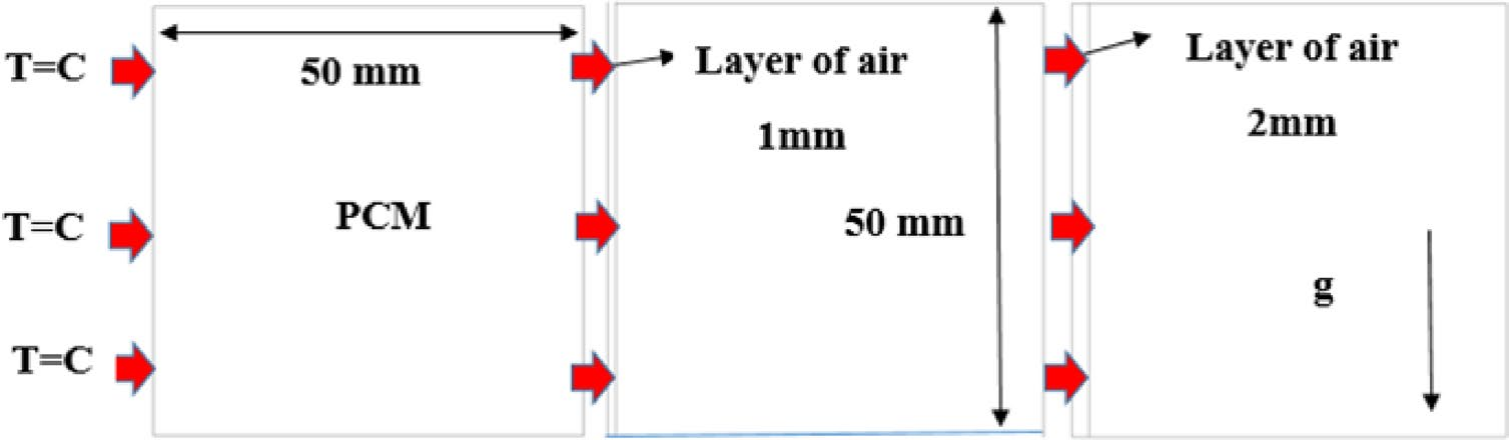
Arrangement of physical model.

A square cell of length filled with phase change materials (PCMs) of dimensions 50 cm is studied first with non-air layer. Subsequently, the presence of an air layer with a thickness of (1, 2) mm are studied to clarify its effect on the melting process and, as in Fig 1.

### Governing equations

The melting process can be accurately predicted when two-dimensional modelling is used through a numerical simulation of a square cell shape. The modelling process is two-dimensional due to its instability, laminar flow, and incompressibility. The procedure necessitates the capture of only the most critical system features. The system requires numerical analysis to produce precise results efficiently, as opposed to the complex three-dimensional simulations that are required. The article provides a comprehensive explanation of the characteristics of phase transitions. The enthalpy-porosity technique, originally developed^30,31^, was used for phase change computational modelling. The technique assumes that the liquid and solid phases of a phase change material (PCM) possess homogeneous and stable properties to achieve thermal equilibrium at their interface. This fundamental assumption includes simplification of simulation variables for enabling precise simulations of phase changes. Also, it was presumed that the system was well isolated, leading to the disregard of viscous dissipation effects and thermal energy loss to the ambient^32–34^. The conservation principles of continuity, momentum, and energy can be stated in terms of these assumptions^35^. The enthalpy method is an effective means of monitoring temperature change in the melting process, enabling accurate measurement of the thermal response and latent heat effect of phase change processes in the PCM. The method enables accurate simulation of the thermal response and energy distribution of the system. The melting is a formidable modelling task because of its nonlinear, unsteady nature and the constantly changing interface between the solid and liquid phases. Only by carefully managing various numerical constraints can stable and steady simulations be achieved since they add complexity to the model. The process is complicated especially because of the phase boundary movement, dissolution rate, and thermophysical properties involved. There are sophisticated numerical techniques that have to be utilized in order to achieve acceptable outcomes. PCMs are defined by a fundamental set of differential equations that maintain the constant values for mass, momentum, and energy because they have the capability to undergo phase transitions as a function of temperature. The thermal performance of a system is primarily estimated using Eqs. (1), (2), and (3), which illustrate the fluid flow and heat transfer within the system. The solutions to the governance equations enable experts to evaluate the distribution of temperatures, the transformation of phases, and the movement of heat through PCM storage systems to optimise their functionality. The equations that regulate the system are listed in the following list^36^:

1 $$\:\frac{\partial\:{\uprho\:}}{\partial\:\text{t}}+\nabla\:.{\uprho\:}\overrightarrow{\text{V}}=0$$

2 $$\:{\uprho\:}\frac{\partial\:\overrightarrow{\text{V}}}{\partial\:\text{t}}+{\uprho\:}\left(\overrightarrow{\text{V}}.\nabla\:\right)\overrightarrow{\text{V}}=-\nabla\:\text{P}+{\upmu\:}\left({\nabla\:}^{2}\overrightarrow{\text{V}}\right)-{{\uprho\:}}_{\text{r}\text{e}\text{f}}{\upbeta\:}\left(\text{T}-{\text{T}}_{\text{r}\text{e}\text{f}}\right)\overrightarrow{\text{g}}-\overrightarrow{\text{S}}$$

3 $$\:\frac{{\uprho\:}{\text{C}}_{\text{p}}\partial\:\text{T}}{\partial\:\text{t}}+\nabla\:\left({\uprho\:}{\text{C}}_{\text{p}}\overrightarrow{\text{V}}\text{T}\right)=\nabla\:\left(\text{k}\nabla\:\text{T}\right)-{\text{S}}_{\text{L}}$$

The incorporation of the damping term ($\:\overrightarrow{\text{S}})$ within the momentum equation is essential to account for the resistance induced by the phase transition process. This term is formulated based on Darcy’s law, which describes the flow behavior in porous media, as referenced in^29^. The damping effect becomes particularly significant in regions where the material transitions between solid and liquid states, influencing the velocity field and overall heat transfer characteristics within the phase change material (PCM). Consequently, this approach ensures a more accurate representation of momentum dissipation during the melting and solidification processes.

4 $$\:\overrightarrow{\text{S}}={\text{A}}_{\text{m}}\frac{{\left(1-{\uplambda\:}\right)}^{2}}{{{\uplambda\:}}^{3}+0.001}\overrightarrow{\text{V}}$$

It has been found from the literature that the constant A_m_ for the mushy region is 105, as given in ref^37,38^. An energy scheme includes a source term to resolve latent heat impact and phase s processes. The liquid fraction of the phase change material (PCM), represented as λ, as shown in reference^39^

5 $$\:{\uplambda\:}=\frac{\varDelta\:\text{H}}{{\text{L}}_{\text{f}}}=\left\{\begin{array}{c}0\:\:\:\:\:\:\:\:\:\:\:\:\:\:\:\:\:\:\:\:\:\:\:\:\:\:\:\:\:\:\:\:\:\:\:\:\:\:\:\:\:\:\:\:\:\:\:\:\:\:\:\:if\:\:T<{\text{T}}_{\text{S}\text{o}\text{l}\text{i}\text{d}\text{u}\text{s}}\\\:1\:\:\:\:\:\:\:\:\:\:\:\:\:\:\:\:\:\:\:\:\:\:\:\:\:\:\:\:\:\:\:\:\:\:\:\:\:\:\:\:\:\:\:\:\:\:\:\:\:\:\:if\:\:T>{\text{T}}_{\text{L}\text{i}\text{q}\text{u}\text{i}\text{d}\text{u}\text{s}}\\\:\frac{\text{T}-{\text{T}}_{\text{S}\text{o}\text{l}\text{i}\text{d}\text{u}\text{s}}}{{\text{T}}_{\text{L}\text{i}\text{q}\text{u}\text{i}\text{d}\text{u}\text{s}}-{\text{T}}_{\text{S}\text{o}\text{l}\text{i}\text{d}\text{u}\text{s}}}\:\:\:\:\:\:\:\:\:\:\:\:\:if\:\:\:{\text{T}}_{\text{S}\text{o}\text{l}\text{i}\text{d}\text{u}\text{s}}<T<{\text{T}}_{\text{L}\text{i}\text{q}\text{u}\text{i}\text{d}\text{u}\text{s}}\end{array}\right\}$$

The specific enthalpy H is the sum of the sensible enthalpy (h) and the latent heat (ΔH).

6 $$\:\text{H}\:=\:\text{h}\:+{\Delta\:}\text{H}$$

Where,

7 $$\:\text{h}={\text{h}}_{\text{r}\text{e}\text{f}}+\underset{{\text{T}}_{\text{r}\text{e}\text{f}}}{\overset{\text{T}}{\int\:}}{\text{C}}_{\text{p}}\:\text{d}\text{T}$$

The source term $\:{\text{S}}_{\text{L}}$ in the energy expression is obtained as follows:

8 $$\:{\text{S}}_{\text{L}}=\frac{{\uprho\:}\partial\:{\uplambda\:}{\text{L}}_{\text{f}}}{\partial\:\text{t}}+{\uprho\:}\nabla\:\left(\overrightarrow{\text{V}}{\uplambda\:}{\text{L}}_{\text{f}}\right)$$

The simulations were conducted using ANSYS/FLUENT 16 with a pressure-based, transient solver in 2D mode. The SIMPLE algorithm was used for pressure-velocity coupling. A second-order upwind scheme was applied for momentum and energy equations. The spatial discretization of the phase change interface was managed using the enthalpy-porosity method. A fixed time step size of 0.5 s was selected after a sensitivity check to ensure both temporal accuracy and computational efficiency. The convergence criteria for each time step were set as follows: 1 × 10⁻⁶ for energy equations, and 1 × 10⁻³ for continuity and momentum equations. The solution was considered converged when all residuals fell below these thresholds. The simulation time was chosen to ensure complete melting across all scenarios.

### Boundary conditions

The study investigates the thermal behavior of a square cell (50 × 50 mm) filled with paraffin wax (RT42), which serves as the phase change material (PCM). A constant temperature of 77 °C is applied to the left vertical wall to simulate the heat source, while the remaining walls (top, bottom, and right) are treated as adiabatic boundaries, assuming no heat loss to the surroundings. The key thermal properties of the paraffin wax (RT42) are detailed in Table 1, providing essential data on factors such as thermal conductivity, latent heat, melting temperature, and specific heat. These properties significantly influence the performance of the PCM, determining how efficiently it absorbs and releases thermal energy during the phase transition.

**Table 1:** The nominal thermal properties of the PCM (RT42)^27^.

Property	RT42
Density, ρ (kg/m^3^)	760
Thermal conductivity, k (W/m.K)	0.2
Specific heat, C_p_ (J/kg K)	2000
Kinematic viscosity, µ (m^2^/s)	5 × 10^− 6^
Thermal expansion coefficient, α (K^− 1^)	0.0005
Latent heat of fusion L (J/kg)	165,000
Melting temperature, T_m_ (°C)	311.5- 3115.5

### Assumptions

Mathematical descriptions of the melting mechanisms inside a square cell take under consideration the following presumptions: Mathematical descriptions of the melting processes within a square cell take under consideration the following presumptions:

1. The concept of melting is depicted in a 2-D form.
2. No heat gains or losses occur from the environment with this type of building design.
3. The flow type in this case is unsteady, laminar and incompressible.
4. The last term under the integration sign represents the viscous dissipation and it is small enough to be omitted.
5. The changes of volume consequent of the transitions of solid-liquid phases are not considered.
6. The thermal properties of the PCMs are considered as constant in its solid and liquid states in the first place.

**Fig. 2:**
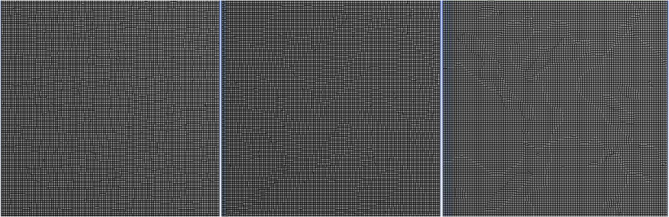
Configuration of mush model.

These assumptions are pivotal in streamlining the mathematical representation of the melting processes, facilitating a more efficient analysis while ensuring that the core physical behaviors remain intact. As depicted in Fig. 2, the mesh distribution for the model under investigation is presented. The mesh is a fundamental component of the numerical analysis, as it defines the spatial resolution of the computational domain. By dividing the domain into discrete cells, it allows for the precise numerical solution of the governing equations. This ensures that the computed temperature, phase change, and heat transfer rates are accurate, ultimately reflecting the true thermodynamic characteristics of the system under study.

### Mesh independence study and validation

**Fig. 3:**
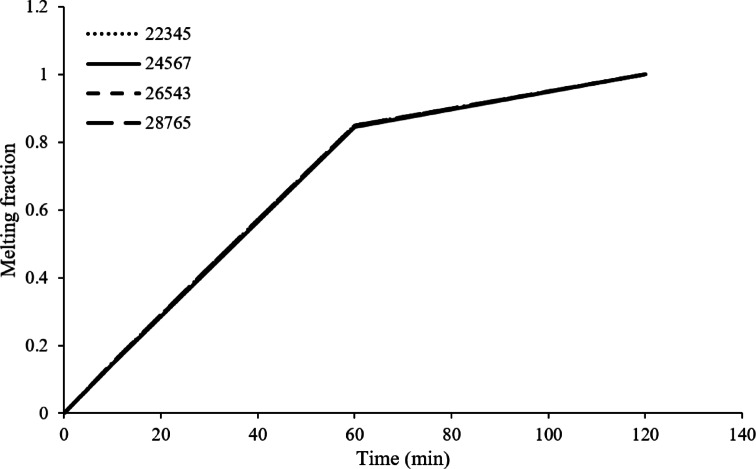
Grid independency test without layer air.

In addition, a thorough convergence analysis was carried out to enhance the credibility and robustness of the computational model in which the degree of influence of mesh resolution is analysed for phase change performance and overall heat transfer characteristics by determining the mesh density at which computational efficiency is combined with necessary resolution requirements for defining critical aspects of phase transition accurately. In this regard, it was found that while refining the mesh produces a more faithful depiction of the phase change front, subsequent effects on the essential parameters are reduced and almost negligible for melting time and temperature distribution. It has been established that a mesh model consisting of 26,543 elements is sufficient to model accurately the phase shifts at reasonable cost. Indeed, this density captured the key temperature and velocity gradients in those locations where phase changes could occur so that predictions remained true to the actual physical phenomena. Completion of the mesh independence study verified that the numeric model met the autonomy criterion, thus assuring that results would not be significantly affected by variations in mesh density. This proved to be a solid foundation for carrying out further simulations and investigations related to the thermal performance of PCMs in various operating conditions. In addition, this study reassured the reliability of the numerical model’s results free from and mesh-related inconsistencies that made it more convincing to evaluate different parameters’ effects, such as temperature gradients, heating methods, and PCM properties on the phase change process. For these reasons, the 22,345-element mesh was suitable for the following simulations as it straddled the balance between efficiency and precision in computation. Computing time was greatly reduced with this mesh as iteration completion was within about 10 min. To further enhance the credibility of the simulation framework, validation at an advanced level, as given in Fig. 3, was carried out. Some new advances were also brought into the simulation code, including improvements in liquid fraction modelling and handling complex geometries. These were in line with an early pioneering contribution by Kean et al.^18^, which went a long way in understanding the science behind heat transfer and fluid dynamics during phase changes of PCMs in square cells.

**Fig. 4:**
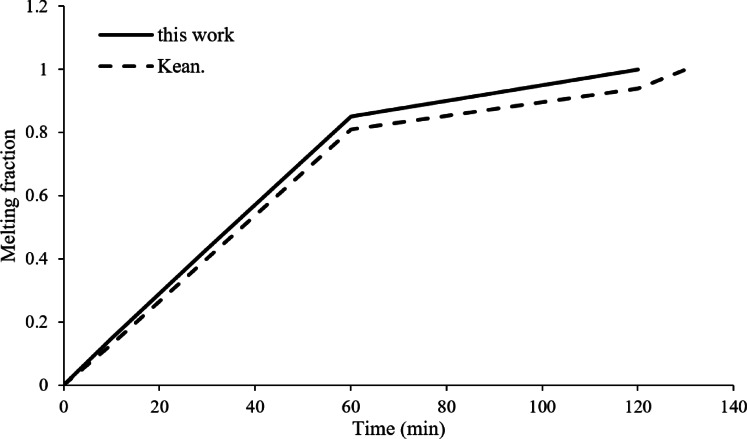
Comparison of this study’s melting fraction vs. operating time to Kean et al.^18^, showing strong agreement (RMSE = 4.2%, max deviation = 6.8%).

A remarkable correlation has been established between results from the improved simulation code and those in Kean et al.‘s work. This comparison reinforces the accuracy and reliability of the newly established simulation framework. There is virtually no distinction between data even when there are considerable dissimilarities in the method and parameters used for computations, as can be seen in Fig. 4. This proves further that the upgraded simulation code is both accurate and efficient in modelling the thermal behaviour of phase change materials.

A quantitative assessment was conducted by comparing the melting fraction evolution in this study with Kean et al.^18^. The Root Mean Square Error (RMSE) was calculated as 4.2%, with a maximum deviation of 6.8% at the mid-melting stage. This close agreement further validates the accuracy of our numerical framework.

## Results and discussion

The melting and solidification processes of phase change materials (PCMs) are of great importance in various thermal energy storage (TES) systems, such as smart building, solar energy system, or even power plant. Geometry and boundary conditions, affecting by exterior factors widely, should be studied in detail to get full understanding of the phase change behavior. In this paper, three different cases of different scenarios were investigated to see how air layer could affect the process of melting PCM: (1) PCM inside the square cell, thereinto has no air layer, (2) PCM with air layer whose thickness is 1 mm, (3) PCM with air layer whose thickness is 2 mm. Through the melting process several time intervals were studied to obtain important variables as temperature distribution, velocity profiles and melting fraction. These results were compared and analyzed to find which differences were significant between the three scenarios. By reviewing these parameters in a comprehensive manner, this study enhances the comprehension of intricate activities happening throughout PCM melting and solidification that will result in the highly effective TES designs for numerous applications.

**Fig. 5:**
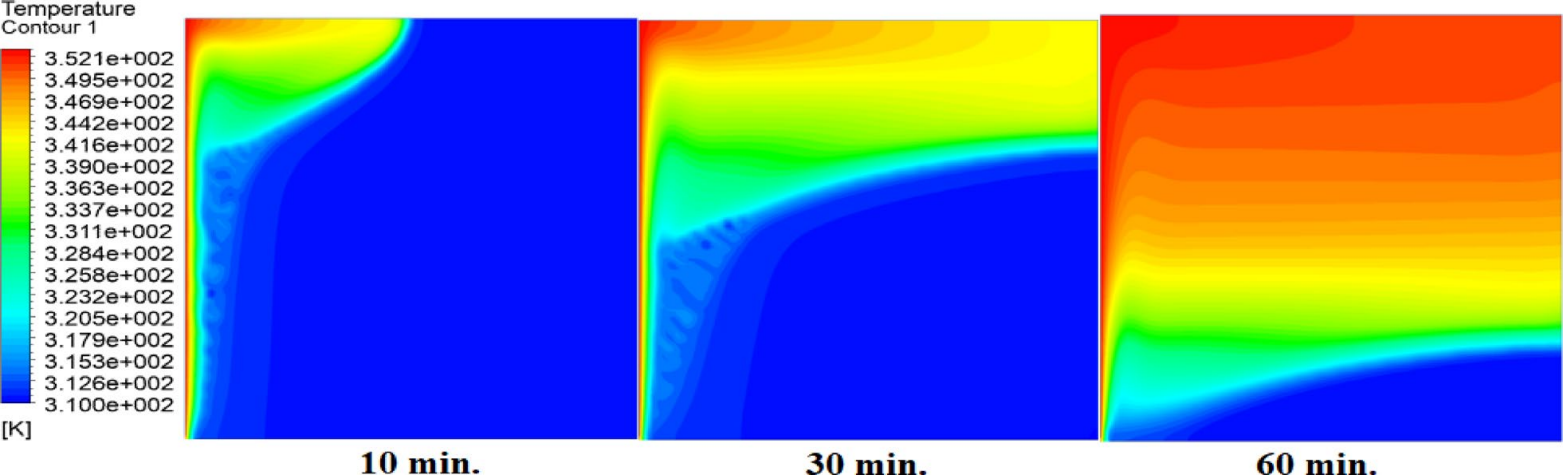
Temperature contours during RT42 melting in a square cell (50 × 50 mm) without an air layer at 10, 30, and 60 min. Left wall heated to 77 °C (red), others insulated. Rapid heat conduction enables complete melting by 60 min.

**Fig. 6:**
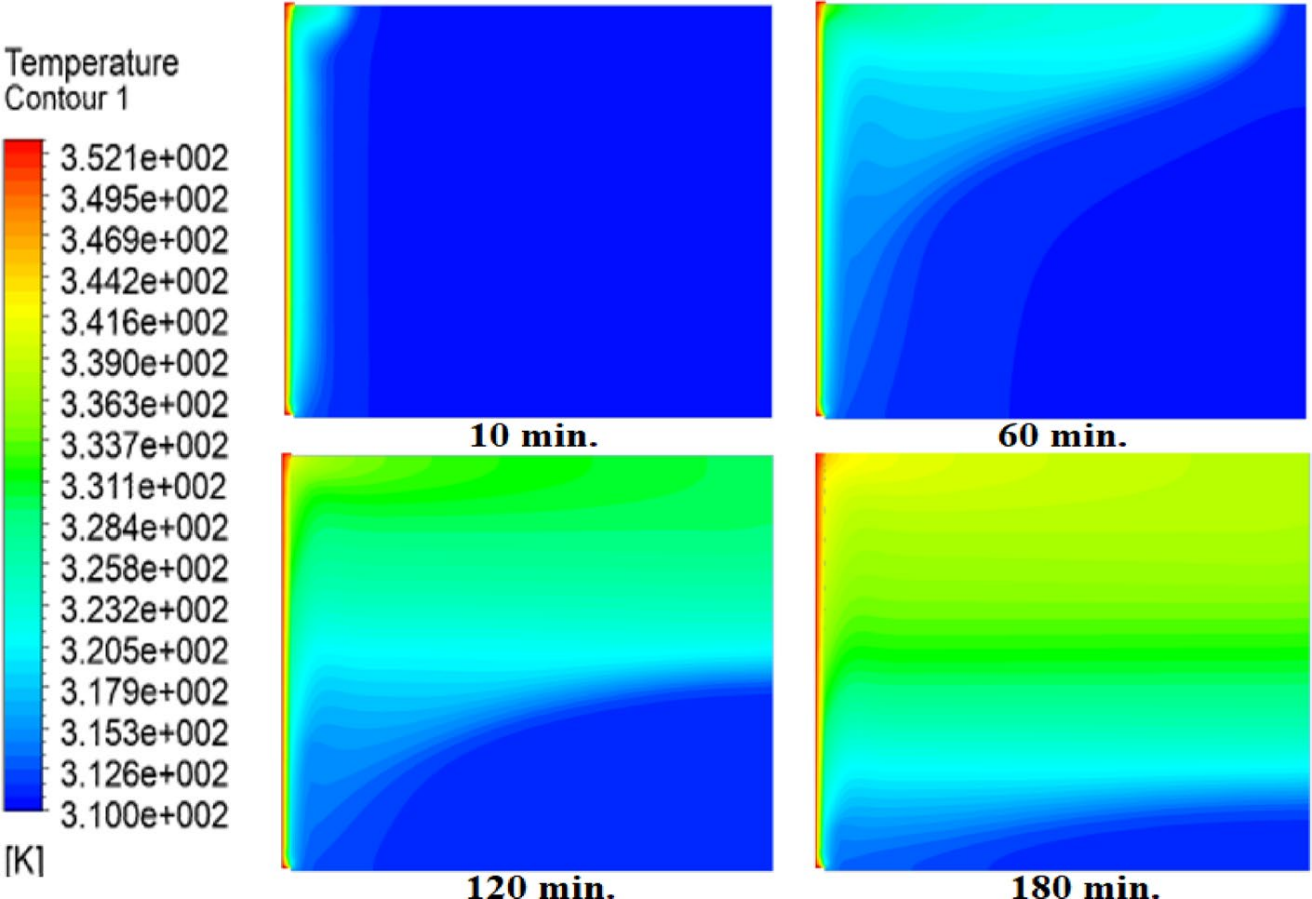
Temperature contours with 1 mm air layer at 10, 30, 60, and 180 min. The air layer reduces heat flux, delaying melting (compare to Fig. 5). Full melting requires 180 min - twice the baseline time.

**Fig. 7:**
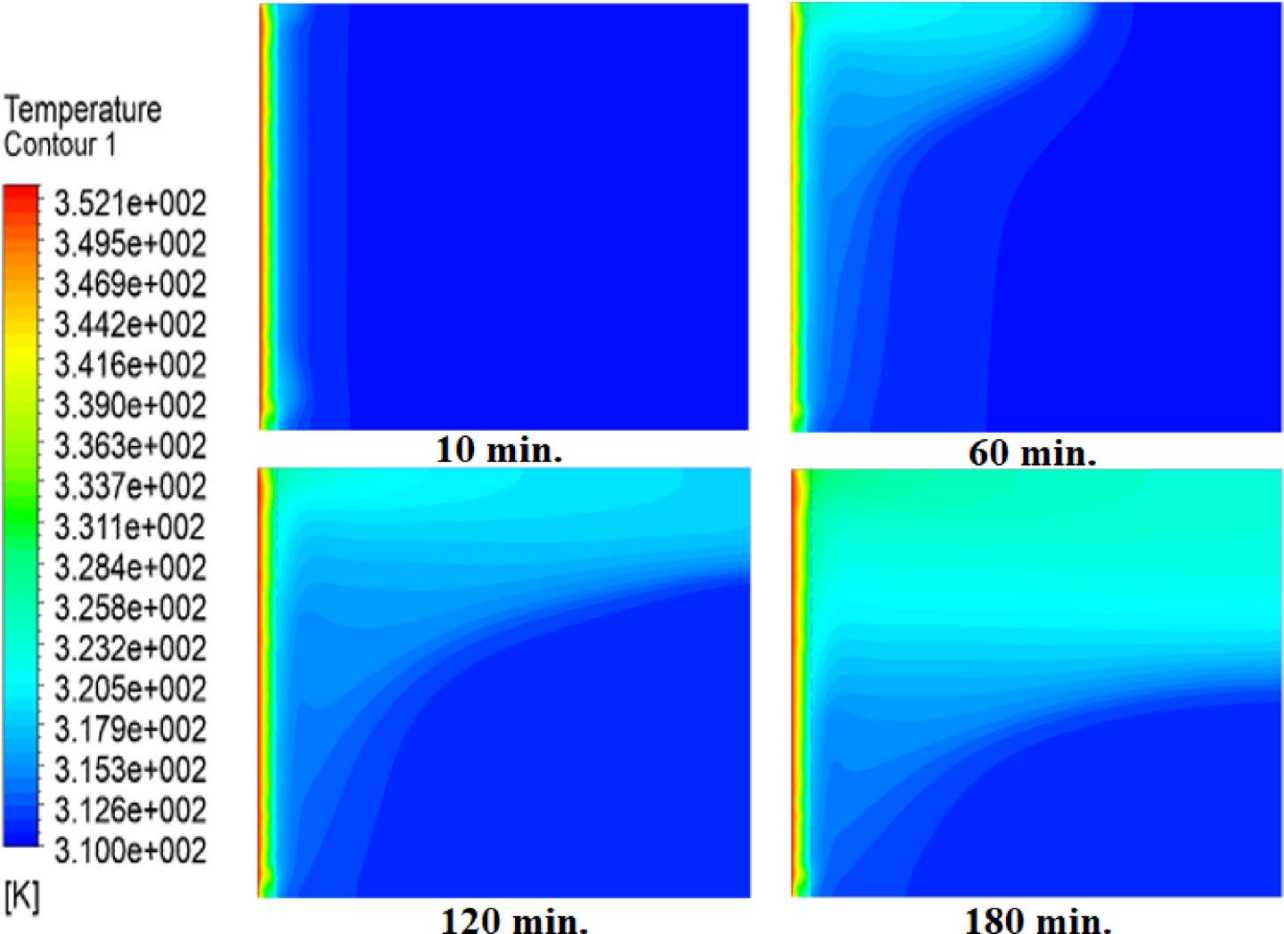
Temperature contours with 2 mm air layer. Thicker air gap further slows heat penetration, tripling melting time to 360 min versus no-air case.

Temperature distributions within the square cell for three cases: without air layer, with 1 mm air layer, with 2 mm air layer are shown in Figs. 5, 6 and 7. In the absence of air layer, PCM temperature near hot wall is increased sharply due to heat conduction, with heat gradually diffuses away from source. A similar trend is noticed with the introduction of air layers; but with a lesser temperature level attained due to the fact that thermal resistance of the air layer interrupts with the heat flux^27^. Additionally, PCM temperatures in regions further from the hot surface initially decrease but eventually rise due to free convection. The intensity of free convection increases in the upper layers, particularly near the hot surface, where higher temperatures are observed in the absence of an air layer. A comparative analysis of the three cases reveals substantial differences in PCM temperature distribution. After 60 min without an air layer, more than 80% of the cell area reaches or exceeds the melting temperature of paraffin wax (315 K). However, with a 1 mm air layer, PCM temperatures drop, requiring 180 min to reach the same levels. This effect is even more pronounced when a 2 mm air layer is introduced, further delaying the heating process.

**Fig. 8:**
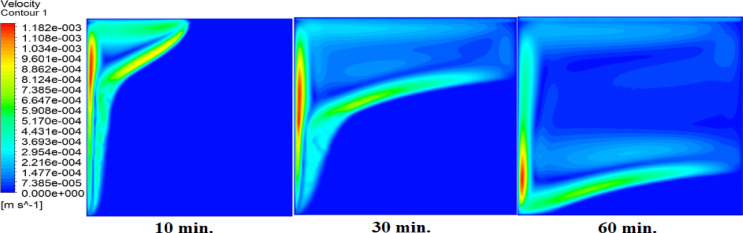
Velocity profiles without air layer. Weak early flows (10 min) strengthen by 30 min as convection dominates, correlating with fast melting in Fig. 5.

**Fig. 9:**
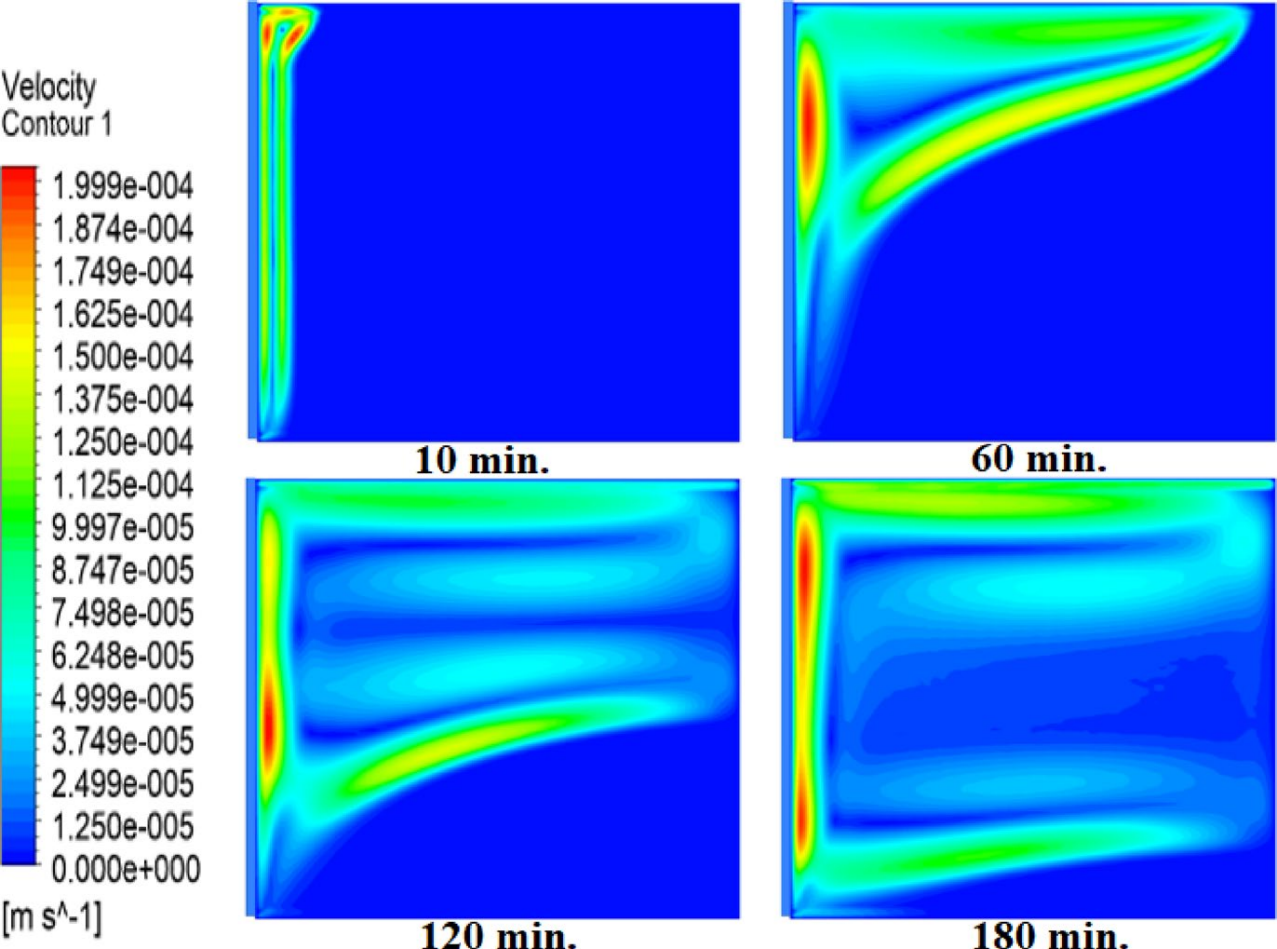
Velocity profiles with 1 mm air layer. Delayed flow development (30 min) confirms suppressed convection, matching the doubled melting time in Fig. 6.

**Fig. 10:**
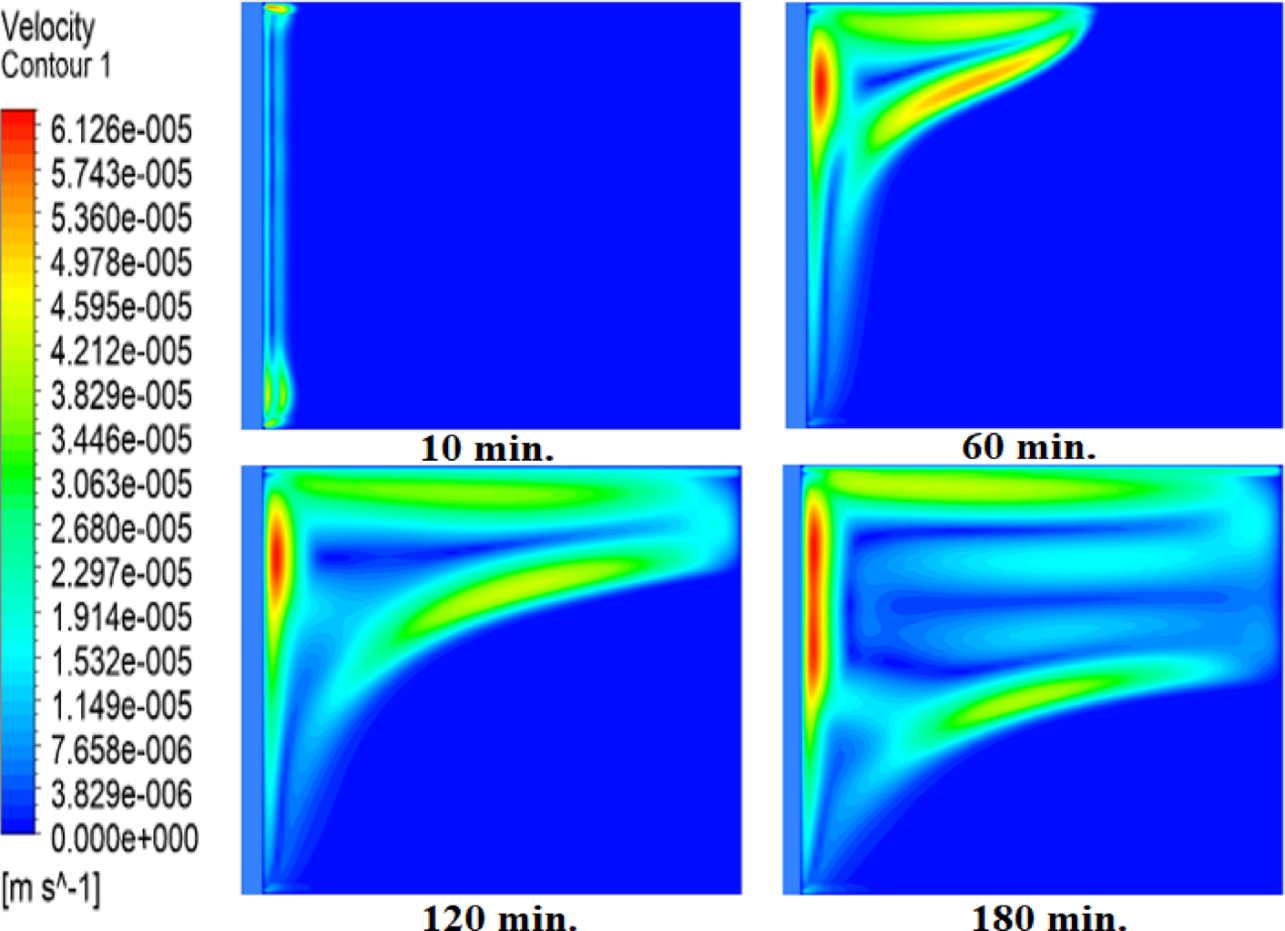
Velocity profiles with 2 mm air layer. Minimal flow at 30 min and weak convection at 240 min explain the tripled melting time.

Figures 8 and 9, and 10 show the velocity profile within the square cell for each of the three examined cases. As the solid paraffin wax (RT42) heats up and melts, velocity profiles develop, and liquid layers form and lift up due to the buoyancy forces created by the temperature gradient. In the case of no air layer, the velocity profiles near the hot surface starts to develop as early as 10 min indicating the start of liquid flow. After 30 min, the velocity profiles penetrate into the intermediate regions, which signifies that an increase in the melting fraction occurs, since more of the PCM has undergone phase transformation. By 60 min, dominant velocity patterns at the bottom, especially near to the hot wall, show that the paraffin is nearly fully melted. But there is a time lag in development of the velocity profile with the existence of an air layer. This delay retards the movement of liquid layers, thus, slows down the entire melting process. With a 1 mm thin air layer, a longer time is required to form the velocity profiles. In fact, the delay is even exacerbated when a 2 mm thick air layer is present, indicating a substantial effect air layers have on the phase change kinetics. These parameters the decisive influence of the air layer thickness on the heat transfer and the phase change rate with thicker air layers having a stronger decelerating effect on the melting process.

To further clarify the convective behavior and heat transfer trends observed in the three scenarios, two key non-dimensional parameters were evaluated: the Rayleigh number and the Nusselt number. The Rayleigh number, representing the ratio of buoyancy to thermal and momentum diffusion, was found to exceed 150,000 in the case without an air layer, indicating strong natural convection consistent with the early and intense flow development. When 1 mm and 2 mm thick air layers were introduced, the effective temperature difference was reduced, leading to lower Rayleigh numbers and weaker convection. Correspondingly, the Nusselt number, which represents the ratio of convective to conductive heat transfer along the heated wall, was observed to decrease from approximately 6.8 (no air layer) to 3.5 and 2.1 for the 1 mm and 2 mm air layers, respectively. These findings confirm that the presence of thicker air layers significantly reduces heat transfer effectiveness, prolongs the melting process, and suppresses free convection.

**Fig. 11:**
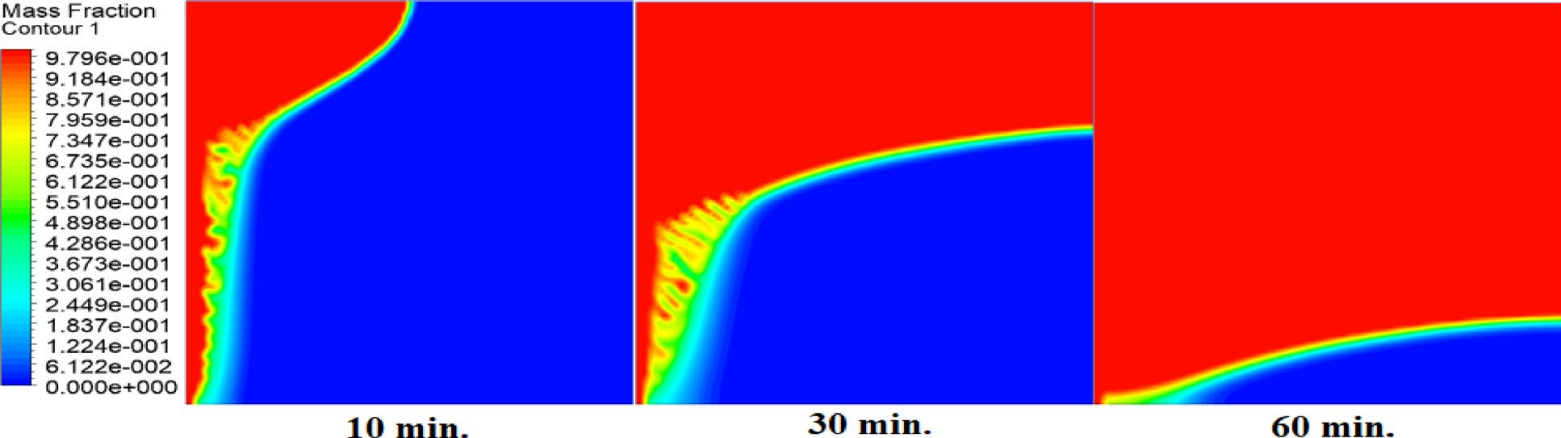
Melting front progression without air layer. Liquid fraction (blue→red) expands rapidly, achieving full melting by 60 min.

**Fig. 12:**
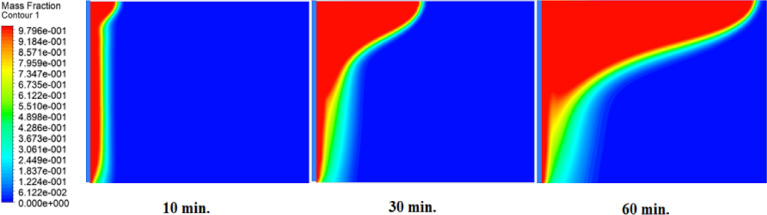
Melting front with 1 mm air layer. Slower interface movement shows solid PCM remaining at 60 min, requiring 180 min for complete melting.

**Fig. 13:**
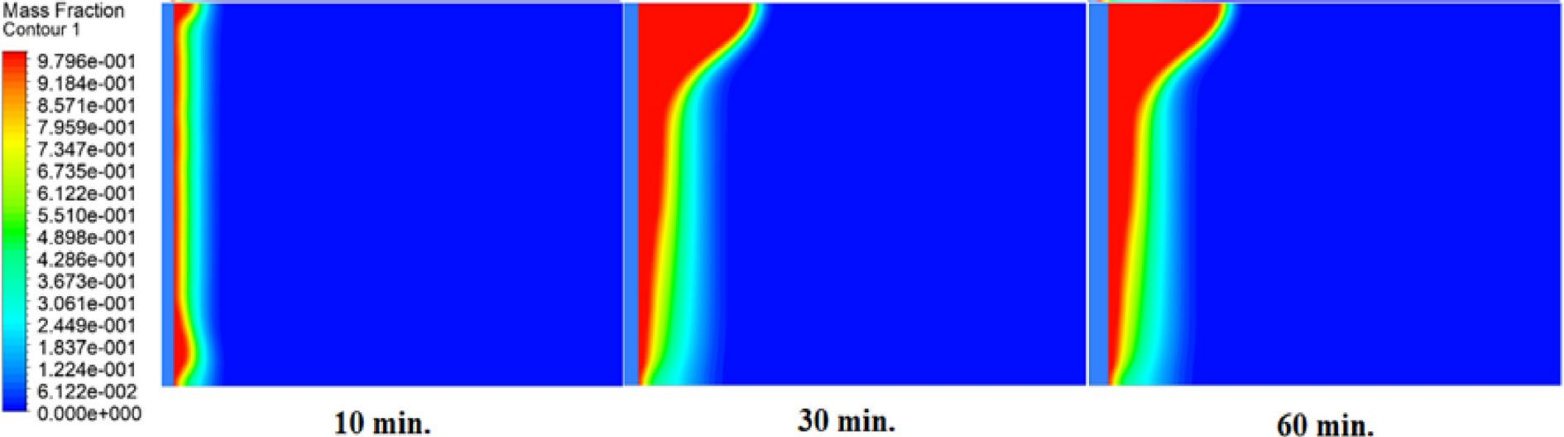
Melting front with 2 mm air layer. Significant solid PCM persists at 240 min, demonstrating the strongest delay effect.

The melting fraction development over a 1-hour period under three conditions are shown in Figs. 11 and 12, and 13, without an air layer, with a 1 mm air layer, and with a 2 mm air layer. Firstly, melting occurs close to the hot surface where the heat transfer is by thermal conductivity. After 10-minute melting fraction is much larger for the case without an air layer, due to the direct heat conduction that efficiently transfer energy into PCM. On the other hand, air layers will impede melting. A 1 mm air layer has a moderate reduction, and the 2 mm air layer has the lowest melting fraction. This is due to the fact that the greater thermal resistance prevents heat flow. By 30 min later, the melting fraction grows further, and the molten PCM commences spreading from the hot surface. At this point, free convection takes place and promotes heat transfer and accelerates melting in more remote areas of the heat source. A heat is still slowed by an air layer, which retards the phase change of both 1 mm and 2 mm air layer cases, with the 2 mm air layer having the largest delay. In the case without an air layer at 60 min, the melting fraction is largely complete and this is indicated by a very short solid-liquid interface. On the contrary, in 1 mm and 2 mm air layer cases, there is a lot of solid PCM, and the solid-liquid interface is longer. This proves that the air layers do block heat transfer.

**Fig. 14:**
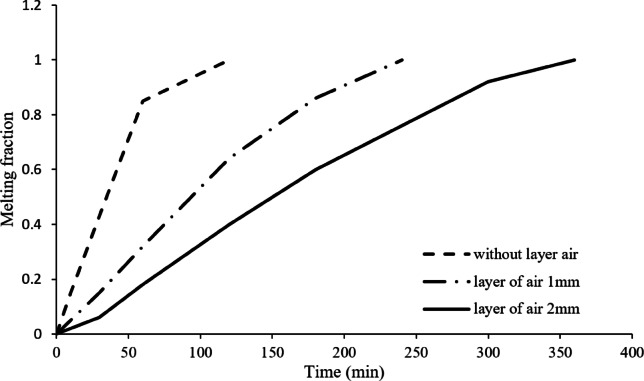
Melting fraction vs. time for all cases. Air layers increase melting time proportionally: 1 mm (2× longer), 2 mm (3× longer).

Figure 14 provides a comprehensive analysis of the complete melting time across the three different scenarios. The results clearly indicate that the melting process is most rapid in the absence of an air layer, followed by the 1 mm air layer case, with the 2 mm air layer case exhibiting the longest melting time. Without an air layer, the entire phase change process is completed in approximately 120 min. However, when a 1 mm thick air gap is inserted, the total melting time is more than doubled to approximately 240 min, signifying a considerable increase in thermal resistance. Besides, when the air layer thickness is increased to 2 mm, the melting time is extended to 360 min and this also clearly show the negative effect of air layers on heat transfer performance. The findings demonstrate that air layers have a considerable impact in slowing down the melting process. Particularly, each extra 1 mm of air layer thickness on the hot left wall of the square cell almost doubles the time need for the PCM to become completely melted. This behavior shows the influence of air gaps on the thermal resistance and phase change kinetics and it demonstrates the significance of the reduction of the air gaps in the applications involving the phase change materials in relation to the enhancement of the energy storage and efficiency of the heat transfer.

Table 2 quantifies the impact of air layers on thermal performance. The 1 mm air layer doubled the melting time (120 → 240 min) and reduced the maximum PCM temperature by 2.8 °C, while the 2 mm layer tripled the melting time (120 → 360 min) with a 5.5 °C temperature drop. The Nusselt number trends further confirm that thicker air layers suppress convection, as Nusselt number decreased from 6.8 (no air) to 2.1 (2 mm air). These metrics underscore the critical trade-off between thermal insulation (from air layers) and energy storage efficiency.

**Table 2:** Comparison of key thermal performance metrics for PCM melting under different air layer configurations.

Case	Complete melting time (min)	Max temperature (°C)	Average Nusselt number
No air layer	120	77.0	6.8
1 mm air layer	240	74.2	3.5
2 mm air layer	360	71.5	2.1

## Conclusions

The performance of the renewable energy applications and the heat recovery systems have a large dependence on the efficiency of the Phase Change Materials (PCMs) used in the thermal energy storage systems. Understanding the effects of natural parameters such as air layers on the melting characteristics of PCMs requires a deep analysis so as to optimize energy storage systems. This study explores the effect of air layers on the melting behavior of the commonly employed paraffin wax (RT42) in a square cell arrangement. The ultimate goal was to study how air layers influence the melting time of paraffin wax (RT42) by computer fluid dynamics (CFD) simulations using software ANSYS/FLUENT 16. The main points from the investigation are as follows:

- The inclusion of a 1 mm thick air layer led to a 100% increase in the total melting time of paraffin wax.
- A 2 mm thick air layer resulted in a 200% increase in the melting time when compared to the case with no air layer.
- The study demonstrated the substantial impact of air layers on the melting process; thicker air layers significantly hindered the melting of paraffin wax.
- The results show how important it is to think about environmental factors, like the thickness of air layers, when planning and designing thermal energy storage systems, especially if you want them to work better.

From an engineering perspective, the findings of this study highlight the importance of controlling air layer formation in PCM-based thermal energy storage systems. Even a thin air gap of 1 mm was shown to double the melting time, while a 2 mm gap tripled it, emphasizing the need to minimize such layers in practical applications. To mitigate these effects, careful attention should be paid during design and manufacturing to ensure proper sealing of enclosures, eliminate air voids, and enhance thermal contact between heating surfaces and PCM. Additionally, the integration of thermally conductive fillers or interface materials may serve to offset the insulating behavior of unavoidable air layers, thereby improving energy transfer efficiency and system performance.

This study demonstrates that air layers critically delay PCM melting, with 1 mm and 2 mm gaps increasing melting time by 100% and 200%, respectively. These results emphasize the need for precise control of air gaps in thermal storage system design.

## Limitations

While this study provides valuable insights into the effect of air layers on PCM melting, several limitations should be considered when interpreting the results. First, the assumption of constant thermal properties for both the PCM and air layer may oversimplify real scenarios where temperature-dependent properties could significantly influence heat transfer rates. For example, variable thermal conductivity in the PCM or air gap might alter the insulating effect, potentially leading to either under- or over-estimation of melting times. Second, the study’s focus on a square cell geometry, while computationally efficient, may not fully represent heat transfer dynamics in other common configurations like cylindrical or spherical containers, where curvature effects could modify convection patterns and melting kinetics. The boundary conditions applied (heated left wall with other sides insulated) also represent an idealized case that neglects practical complexities such as ambient temperature fluctuations, multi-directional heat sources, or convective losses to the environment. Additionally, the use of RT42 paraffin wax as the sole PCM limits the generalizability of findings, as other materials like salt hydrates, fatty acids, or composite PCMs with different thermal conductivities, phase change temperatures, or viscosity characteristics may respond differently to air layer effects. The static treatment of the air layer further overlooks potential dynamic behaviors, such as natural convection within the gap or airflow-induced disturbances, which could locally enhance or disrupt heat transfer in real-world applications.

While these simplifications were necessary to isolate the fundamental impact of air layers, future work should address these limitations through more comprehensive models incorporating material property variability, alternative geometries, and realistic boundary conditions to improve the practical relevance of the findings.

## Future work

- Future studies should investigate the effect of air layers on the PCM melting process in different geometrical forms, including cylindrical or spherical containers, to understand the wider relevance of the conclusions. The curvature effects in these configurations may modify the convection patterns and phase change behavior compared to square cells.
- Examining the effects of several boundary conditions - such as changing the heat source’s location (e.g., applying heat flux from the top or bottom) and including convective boundary conditions - may help to develop a more complete knowledge of the melting dynamics. The interaction between air layers and varied heating directions remains unexplored.
- Alternative PCMs should be incorporated in future research to include many kinds of PCMs with diverse thermal characteristics. This will enable the determination of the generalizability of the results and identification of suitable materials for certain applications, particularly those with different phase change temperatures or viscosity properties.
- The impacts of outside environmental elements, like different ambient temperatures and airflow surrounding the container, would help to more fairly evaluate the thermal storage unit’s performance. Such practical considerations are crucial for real-world implementation.
- Investigating the effects of dynamic air layers - those affected by external airflow or those varying in thickness over time - may provide understanding of how to maximize thermal resistance and enhance heat transfer efficiency. The current study considered only static air layers.
- Enhanced Heat Transfer Techniques: Researching the employment of fins, nanoparticles, or other heat transfer enhancing techniques in combination with air layers might expose synergies that raise the general efficiency of thermal energy storage systems. The combined effects of these methods with air gaps remain unknown.

Through addressing these research directions, scientists may build a stronger knowledge of the interactions between PCMs, air layers, and heat transfer processes, therefore enabling more efficient and effective thermal energy storage methods for renewable energy applications.

## Data Availability

The datasets used and/or analysed during the current study available from the corresponding author on reasonable request.
